# Are selected IL-1 polymorphisms and selected subgingival microorganisms significantly associated to periodontitis in type 2 diabetes patients? a clinical study

**DOI:** 10.1186/s12903-015-0132-5

**Published:** 2015-11-14

**Authors:** Herbert Deppe, Thomas Mücke, Stefan Wagenpfeil, Marco Kesting, Julia Karl, Sebastian Noe, Anton Sculean

**Affiliations:** 1 Department of Oral and Maxillofacial Surgery, Technical University of Munich, Klinikum rechts der Isar, Ismaninger Strasse 22, Homburg Saar, D-81675 Munich Germany; 2 Institute for Medical Biometry, Epidemiology and Medical Informatics, University of Saarland, Homburg Saar, Germany; 3 Department of Internal Medical Department II, Technical University of Munich, Klinikum rechts der Isar, Homburg Saar, Germany; 4 Department of Periodontology, University of Berne, Berne, Switzerland

**Keywords:** Diabetes, Periodontitis, Metabolic control, *IL1A*, Microbiology

## Abstract

**Background:**

We evaluated the periodontal conditions in patients with type 2 diabetes mellitus compared to metabolically healthy controls, and determined whether periodontal interleukin genotypes and microorganisms differed between participants with and without type 2 diabetes mellitus.

**Methods:**

From April 2011 to July 2012, we prospectively enrolled healthy controls and patients with type 2 diabetes mellitus. Evaluation included assessment of medical and periodontal findings. We also recorded the presence of several interleukin gene variants and specific microorganisms, both available through commercially available diagnostic kits. Statistical significance was tested by the chi-square test and student’s *t-*test.

**Results:**

We enrolled 52 patients with type 2 diabetes mellitus and 52 healthy controls. Compared with controls, periodontal disease was significantly more severe in patients with type 2 diabetes mellitus for the following: plaque index, bleeding on probing, pocket probing depth, clinical attachment loss, severe periodontal destruction (i.e., clinical attachment loss ≥ 5 mm), and number of teeth. However, statistical analysis failed to detect significant differences with respect to the periodontal-related interleukin genotypes (*p* ≥ 0.58) or the selected oral microbiota (*p* ≥ 0.15).

**Conclusion:**

Based on these results, it may be assumed that chronic periodontitis in patients with type 2 diabetes mellitus is most strongly associated with inadequate oral hygiene. Periodontal interleukin genotypes and differences in oral microbiota seem to play a subordinate role.

## Background

Periodontitis is a common chronic disease of tooth-supporting tissues caused by bacterial deposits that accumulate on the tooth surface and form dental plaque [1]. Over recent years, there has been increased interest in determining whether a link exists between periodontal health and overall health or disease [1–4]. As a local oral inflammatory disease, periodontitis may induce and perpetuate systemic inflammation that aggravates cardiovascular disease, pulmonary disease, rheumatoid arthritis, and diabetes mellitus (DM) [1].

The most common human endocrine disease, DM is a metabolic disorder characterized by chronic hyperglycemia [5]. Type 2 DM (T2DM) is the most prevalent type, occurring in 90 %–95 % of all patients with DM [6, 7]. Poor glycemic control in patients with T2DM leads to prolonged blood glucose elevations that damages blood vessels and causes a plethora of associated complications. These include atherosclerosis, myocardial infarction, retinopathy, nephropathy, neuropathy, delayed wound healing, and an increased risk of infection [8].

Several studies have investigated the association between periodontal disease and DM [9], generally concluding that patients with T2DM had more extensive periodontal disease than nondiabetic controls [10]. Although oral hygiene seems to be key to the progression of severe periodontal destruction [11], it is equally plausible that other intrinsic factors are influential. Accordingly, recent literature has produced conflicting data as to whether patients with DM and comorbid chronic periodontitis have an altered subgingival microbiota compared with healthy controls [12]. In addition, the influence of a genetic polymorphism in the interleukin 1 gene cluster, especially the interleukin 1 beta (*IL1B*) (+3954) genotype, remains a matter of debate [11, 13].

From a therapeutic perspective, it would be interesting to know which variables contribute most significantly to periodontitis in T2DM. It would be equally useful to know whether these patients are affected by periodontal-related interleukin genotype polymorphisms and destructive oral microbiota significantly more often than healthy controls. Detection of such genetic and microbial differences might have a major impact on periodontal treatment in patients with T2DM.

The primary aim of this study was to evaluate the differences in periodontal conditions and influencing variables, such as oral hygiene, between patients with T2DM and healthy controls. In addition, we aimed to evaluate whether there were significant differences in the presence of polymorphisms in the periodontal interleukin 1 genotype and oral microbiota between patients with T2DM and healthy controls.

## Methods

### Study design

This prospective clinical study took place from April 2011 to July 2012 in the outpatient department for internal medicine at the Technical University of Munich. Patients with T2DM diagnosed for at least one year, aged at least 18 years, and attending the internal medicine outpatient department of our university hospital were approached and informed of the study’s aims. For comparison, a control group was recruited from among metabolically healthy patients attending the same department. The study was performed in accordance with the ethical standards of the Declaration of Helsinki and approved by the institutional ethics board of the Technische Universität München, Klinikum rechts der Isar.

### Participants

We included dentate patients who had at least eight remaining teeth (i.e., two molars, two premolars, and four anterior teeth per jaw, free of prosthetic crown restoration) [8]. Patients were excluded if they had fewer teeth, had used antibiotics in the past three months, or were treated circumferentially with prosthetic bridge or crown restorations [8]. Additionally, were also excluded patients with removable dental restorations and those who had attended periodontal therapy or dental prophylaxis appointments within the last six months [8]. With respect to general health, patients were excluded if they had undergone head and neck radiotherapy, systemic bisphosphonate therapy, or were suffering from heart defects or immunosuppression (e.g., corticoid therapy or HIV infection). Finally, pregnant or lactating women were also excluded. To eliminate bias, a single dentist (JK), who was blinded to whether a patient had diabetes, performed all the dental exams.

### Sample size calculation

The sample size was calculated by reference to pocket probing depth (PPD) and clinical attachment loss (CAL) in patients with and without periodontitis [14, 15] in the control and T2DM groups. With respect to PPD, a sample size of 44 per group was needed to give a 90 % power to detect a mean difference of 0.5 mm (ratio of standard deviation: 0.8) using a two group Satterthwaite *t*-test with a 0.05 two-sided significance level. With respect to CAL, a sample size of 48 per group was needed to give a power of 80 % to detect a mean difference of 0.9 mm (ratio of standard deviation: 0.9).

### Variables

#### Medical evaluation

All medical information could be extracted from the patient’s medical charts. Using standardized forms, age, gender, body mass index (BMI) [16], and smoking status (yes/no) were recorded from these charts. Metabolic control was determined by recording the fasting glucose and the glycated hemoglobin (HbA1c) levels from the charts of participants. Finally, we recorded what therapies and medications the patients and controls were receiving.

#### Periodontal evaluation

Periodontal evaluation took place under standardized conditions with the same examiner (JK). Oral hygiene was measured for each tooth according to the plaque index (PI) [17], as follows. A score of 0 was given for no plaque. A score of 1 for a film of plaque that was adherent to the free gingival margin and adjacent area of the tooth. Moderate accumulation of soft deposits within the gingival pocket, or of the tooth and gingival margin, but that were macroscopically visible, were assigned a score of 2. Abundant soft matter within the gingival pocket and/or on the tooth and gingival margin was assigned a score of 3. For assessment of bleeding sites indicative of local inflammation, a modified bleeding-on-probing (BOP) index was scored as a percentage of sites that showed bleeding 30 s after gentle probing of the bottom of the pockets [18, 19].

The PPD was measured to the nearest millimeter on all teeth, except the third molars, and on four sites (mesio-vestibular, disto-vestibular, mesio-oral and disto-oral) from the gingival margin to the base of the pocket or crevice with a WHO probe (Morita, Kyoto/Japan) [20]. In each of these sites, the CAL was also measured from the cemento-enamel junction to the bottom of the pocket [5]. For each individual, the periodontal condition was characterized by its severity and extent. Severity was described for all the individual sites as a whole and was categorized as severe in case of a CAL ≥ 5 mm [11, 21]. Extent was characterized as localized if ≤30 % of the sites were affected and generalized if >30 % of the sites were affected withCAL ≥ 1 mm [21].

In addition, the total number of teeth was recorded. Finally, tooth mobility was assessed manually and classified into four grades: 0, normal, no mobility; 1, minimal mobility; 2, visible mobility (≤1 mm); and 3, marked instability (>1 mm) [22].

The examiner demonstrated reproducibility of periodontal parameter measurements. Prior to the study, both the clinical examiner (JK) and an experienced clinician (HD) assessed the PI, BOP index, PPD, CAL, and tooth mobility in a sample of 10 subjects. Cohen’s kappa coefficients were calculated for each parameter, and ranged between 0.71 and 0.92, which showed good inter-rater reliability. Three days later, intra-rater reliability was confirmed in the same 10 subjects, producing kappa values between 0.81 and 0.89.

#### Genetic diagnosis

We used the commercially available diagnostic test, Geno Type®IL-1 (Hain Lifescience, D–Nehren). This produces four different genetic patterns by DNA hybridization to allow evaluation of hereditary propensity to infection, which we labeled groups A to D based on positivity for interleukin 1 alpha (*IL1A*), *IL1B,* and interleukin 1 receptor antagonist (*IL1RN*) genotypes. In group A, the genotype was not positive for either *IL1A, IL1B,* or *IL1RN*. In group B, the genotype was negative for *IL1A* and *IL1B,* but positive for *IL1RN*. In group C, the *IL1A* and *IL1B* genotypes were positive, but *IL1RN* was negative. Finally, in group D, the genotype was positive for *IL1A, IL1B,* and *IL1RN*. Clinically, groups C and D were expected to be more likely to develop periodontitis than were groups A and B. Sampling of oral mucosal cells was performed using a sterile swab that was placed into a container and sent to the Hain Lifescience Company for analysis.

#### Microbiology

It was demonstrated by Socransky et al. in 1998 that bacterial species exist in complexes in subgingival plaque, most probably due to synergistic effects [23, 24]. Five major clusters were consistently observed, the so-called red, orange, green, yellow, and purple clusters. The commercially available Micro-IDent®plus Test (Hain Lifescience, D–Nehren) allows detection of eleven periodontal pathogenic bacteria using polymerase chain reaction. Accordingly, the test uses a small and limited spectrum of selected microorganisms. It describes eight profiles of complexes with respect to possible antibiotic therapies. Profile 1 detects microbiota of the *Aa-*complex; Profile 2, of the red and orange complex; and Profile 3, of the red and orange complexes plus *Peptostreptococcus micros*. In turn, Profile 4 detects bacteria of the orange complex; Profile 5, of the green complex; and Profile 6, a combination of the *Aa*- and green complexes. Profile 7, similar to Profile 8, detects a combination of the *Aa*-complex, green complex, and the red and orange complexes, with the difference being that the concentration of *Peptostreptococcus micros* in Profile 7 does not require therapy.

In each patient, subgingival plaque samples were obtained using sterile paper points, which were placed with sterile forceps for 10 s in the deepest periodontal pocket of each sextant of the jaw. Cotton rolls were used to isolate the sampling area from saliva. Paper points were placed into a container and sent to the Hain Lifescience Company for analysis.

### Statistical analysis

The data were analyzed using IBM SPSS for Windows, Version 19.0 (IBM Corp., Armonk, NY, USA), and a P-value less than 0.05 was considered to indicate statistical significance in all analyses. Data of the clinical parameters are presented as means ± standard deviations, or as continuous variables and proportions. The statistical significance of difference in proportions was tested by the chi-square test. Statistical testing between continuous variables was performed using student’s *t* tests. Correlations between medical and periodontal findings were analyzed by statistical means, using BOP, CAL ≥ 5 mm, and number of teeth [11].

## Results

### Patients

**Fig. 1:**
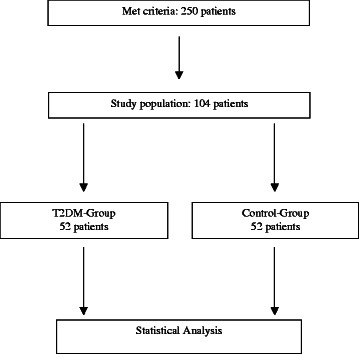
Flowchart showing the distribution of patients included into the study

Among the patients invited to participate in this study, 250 fulfilled the inclusion criteria (178 patients with T2DM and 72 controls). In accordance with the power calculation, we obtained written informed consent for dental evaluation from 52 patients with type 2 DM and 52 patients with healthy metabolisms. All participants were white Caucasians. According to the chart reviews, both groups were comparable with respect to all diseases, except for the implantation of cardiac pacemakers (*p* = 0.04) and presence of hepatitis B and C (*p* = 0.03), which were significantly more common in the T2DM group (Fig. 1 and Table 1). Statistical analysis revealed no significant difference between the groups in age, gender, or smoking habits, but the T2DM group did have significantly higher mean impaired fasting glucose values compared with controls. Moreover, mean HbA1c and BMI values were significantly higher in the T2DM group compared with the control group. The mean duration of diabetes was 8.9 ± 6.3 years.

**Table 1:** Baseline characteristics of study participants (*N* = 104)

Variable	T2DM Group (*n* = 52)	Controls (*n* = 52)	*P*-value
Subjects (male/female; N)	36 / 16	27 / 25	0.07
Age (years; mean ± SD)	63.4 ± 13.6	58.8 ± 13.5	0.08
Current smoker (n, %)	9 (17.3 %)	6 (11.5 %)	0.40
Former and never smokers (n, %)	43 (82.7 %)	46 (88.5 %)	0.81
Fasting glucose (mg/dl, mean ± SD)	179.4 ± 80.3	100.8 ± 14.6	<0.001
HbA1c level (%, mean ± SD)	7.1 ± 1.7	5.4 ± 0.5	<0.001
BMI (kg/m^2^, mean ± SD)	28 ± 5.9	25.8 ± 4.8	0.039

### Periodontal findings

Plaque index values indicated significantly better oral hygiene in the control group compared with the T2DM group. According to the BOP index, the T2DM group demonstrated significantly more bleeding sites than the control group. Moreover, these patients showed significantly higher mean PPD and CAL values, as well as significantly more severely affected periodontal pockets (CAL ≥ 5 mm), compared with controls. Similarly, more patients in the T2DM group suffered from generalized periodontitis and visible mobility or marked instability of teeth (scores 3 and 4). In addition, the T2DM group had significantly less teeth than the control group, but there was no statistically significant difference between the groups with respect to localized periodontitis or tooth mobility (scores 1 and 2) (Table 2).

**Table 2:** Periodontal findings and monitored parameters in the study participants (*n* = 104)

Variable	T2DM Group (*n* = 52)	Controls (*n* = 52)	*P*-value
PI (score 0–3) (mean ± SD)	1.6 ± 0.2	1.2 ± 0.1	<0.001
BOP (%) (mean ± SD)	7.0 ± 6.9	2.4 ± 2.8	<0.001
PPD (mm) (mean ± SD)	4.6 ± 0.9	4.0 ± 0.8	<0.001
CAL (mm) (mean ± SD)	7.4 ± 1.6	6.5 ± 1.5	0.003
Severe periodontitis (CAL ≥ 5 mm) n (%)	38 (73.1 %)	19 (36.5 %)	<0.001
Generalized periodontitis n (%)	10 (19 %)	1 (2 %)	0.004
Localized periodontitis n (%)	38 (73 %)	36 (69 %)	0.66
Number of Teeth (mean ± SD)	16.8 ± 8.7	21 ± 8.5	0.014
Mobility Grade 0 and 1, n (%)	41 (78.8 % )	49 (94.2 %)	0.34
Mobility Grade 2 and 3, n (%)	11 (2.11 %)	3 (5.8 %)	0.044

Tables 3 and 4 demonstrate that the estimated periodontal parameters were related to age in the T2DM group, but not in the control group. For patients with T2DM who were older than 45 years, periodontal disease was more severe. With respect to good glycemic control (HbA1c < 7), patients with T2DM had a significantly lower mean BOP index than patients with poor glycemic control (HbA1c **≥** 7). However, comparison of HbA1c levels with severe periodontal destruction (CAL ≥ 5 mm) and number of teeth did not reach statistical significance in the T2DM group. Among the control group, no individuals had a HbA1c level ≥ 7; accordingly, statistical analysis could not be performed. With respect to smoking habits, there were no significant differences in the three estimated parameters for current smokers versus former and never smokers in the T2DM group. Only severe periodontal destruction (CAL ≥ 5 mm) differed significantly between current and formerly/never smokers in the control group. Tables 3 and 4 also demonstrate that among the estimated periodontal parameters, BOP and severe periodontal destruction (a CAL ≥ 5 mm), but not the number of teeth, were significantly related to the patients’ oral hygiene levels (PI ≥ 1) in both the T2DM and control groups.

**Table 3:** Correlation of medical and periodontal findings in patients with T2DM

Variable	BOP (%) Mean ± SD	*P*-value	CAL ≥ 5 mm % (n)	*P*-value	Number of teeth Mean ± SD	*P*-value
Age ≥ 45 years	6.47 ± 7.1 (*n* = 43)	0.18	87.8 (*n* = 36/41)	<0.001	15.65 ± 8.6 (*n* = 43)	<0.001
Age < 45 years	3.25 ± 0.35 (*n* = 9)		18.2 (*n* = 2/11)		27.00 ± 1.41 (*n* = 9)	
HbA1c ≥ 7 %	10.81 ± 8.2 (*n* = 18)	<0.001	83.3 (*n* = 15/18)	0.31	14.00 ± 8.8 (*n* = 18)	0.19
HbA1c < 7 %	3.65 ± 4.30 (*n* = 34)		70.58 (*n* = 24/34)		17.38 ± 8.72 (*n* = 34)	
Current smokers	7.42 ± 6.56 (*n* = 9)	0.77	66.6 (*n* = 6/9)	0.17	14.33 ± 10.6 (*n* = 9)	0.52
Former and never smokers	6.67 ± 7.26 (*n* = 43)		74.4 (*n* = 32/43)		16.43 ± 8.6 (*n* = 43)	
PI ≥ 1	7.5 ± 7.2 (*n* = 43)	0.004	83.3 (*n* = 35/42)	<0.001	16.3 ± 8.7 (*n* = 43)	0.73
PI < 1	0.1 ± 0.1 (*n* = 9)		0.30 (*n* = 3/10)		15.3 ± 1.5 (*n* = 9)

**Table 4:** Correlation of medical and periodontal findings in controls

Variable	BOP (%) Mean ± SD	*P*-value	CAL ≥ 5 mm % (*n*)	*P*-value	Number of teeth Mean ± SD	*P*-value
Age ≥ 45 years	2.35 ± 3.00 (*n* = 43)	0.84	33.3 (*n* = 14/42)	0.41	20.00 ± 8.84 (*n* = 43)	0.06
Age < 45 years	2.56 ± 2.01 (*n* = 9)		20.0 (*n* = 2/10)		25.89 ± 4.73 (*n* = 9)	
HbA1c ≥ 7 %	-	-	-		-	
HbA1c < 7 %	2.50 ± 3.05 (*n* = 52)		38.46 (*n* = 20/52)		20.20 ± 8.99 (*n* = 52)	
Current smokers	1.67 ± 1.97 (*n* = 6)	0.13	83.3 (*n* = 5/6)	0.016	18.67 ± 10.19 (*n* = 6)	0.48
Former and never smokers	2.48 ± 2.93 (*n* = 46)		32.6 (*n* = 15/46)		21.32 ± 8.38 (*n* = 46)	
PI ≥ 1	2.8 ± 2.6 (*n* = 48)	0.045	87.2 (*n* = 41/47)	0.008	21.4 ± 8.37 (*n* = 48)	0.84
PI < 1	0.1 ± 0.1 (*n* = 4)		40.0 (*n* = 2/5)		20.5 ± 11.8 (*n* = 4)	

### Genetic and microbiologic findings

According to the four genetic groups, A to D, the chi-square-test demonstrated no statistically significant differences between the T2DM and control groups (*p* ≥ 0.58). Similarly, statistical analysis failed to detect significant differences between the groups in terms of the selected oral microbiota (*p* ≥ 0.15) (Tables 4 and 5). Although concentrations of bacteria generally did not differ significantly between both groups, those of *F. nucleatum* (*p* = 0.029) and *E. corrodens* (*p* = 0.042) were higher in the control group, while that of *C. rectus* was higher in the T2DM group (*p* = 0.031).

**Table 5:** Genetic and microbiologic findings

Parameter (n) %	*T2DM Group n* = 52	*Controls n* = 52	*P*-value
Genetic group A	19 (36.5 %)	16 (30.8 %)	0.58
Genetic group B	16 (30.8 %)	19 (36.5 %)	0.58
Genetic group C	9 (17.3 %)	8 (15.4 %)	0.79
Genetic group D	8 (15.4 %)	9 (17.3 %)	0.79
Microbiological Profile 1	1 (1.9 %)	0 (0 %)	0.31
Microbiological Profile 2	26 (50 %)	19 (36.5 %)	0.17
Microbiological Profile 3	5 (9.6 %)	6 (11.5 %)	0.75
Microbiological Profile 4	2 (3.8 %)	0 (0 %)	0.15
Microbiological Profile 5	0 (0 %)	2 (3.8 %)	0.15
Microbiological Profile 6	0 (0 %)	0 (0 %)	-
Microbiological Profile 7	3 (5.8 %)	3 (5.8 %)	1
Microbiological Profile 8	1 (1.9 %)	1 (1.9 %)	1
No Microbiological Profile 1–8	14 (26.9 %)	21 (40.4 %)	0.15

## Discussion

Periodontitis and DM both place enormous costs on the public health care system [25, 26]. Therefore, clinical studies that address both diseases are urgently needed. Accordingly, we aimed to compare the periodontal conditions between patients with T2DM and healthy controls, with a specific interest in the roles of differences in specific genetic polymorphisms and oral microbiota, because such differences might affect periodontal treatment in patients with T2DM.

In this study, 72.2 % of controls (52 of 72) participated in the study, compared with 29.2 % of patients with T2DM (52 of 178), which is consistent with the known literature [11]. With respect to age, gender, and smoking habits, there were no statistically significant differences between patients and controls, which is also consistent with the literature [4, 8, 18, 27]. However, the percentage of smokers was below the German mean (27.6 %), which might be attributable to the relatively small sample size [28].

### Periodontal findings

We assessed periodontal status without x-rays because of the poor ability of this imaging modality to reflect the real periodontal situation [29, 30]. Indeed, our clinical results showed that periodontal disease was significantly worse in patients with T2DM on most parameters (PI, BOP, PPD, CAL, CAL ≥ 5 mm, number of teeth, and scores of 2 and 3 on tooth mobility) (Table 2). These findings are consistent with the data of other studies [4, 31, 32]. Also similar to our results, patients with T2DM in another clinical study had 18 ± 7 teeth, which compared with 22 ± 5 teeth in their control group [33].

With respect to age, only patients with T2DM who were older than 45 years showed significantly worse periodontal disease, but this difference did not persist when older and younger controls were compared (Tables 3 and 4). Accordingly, we concluded that age was not a significant influencing factor in this study. Similarly, previous studies of patients with DM have failed to demonstrate a positive correlation between the periodontal state and age, instead suggesting that age was much less important when oral hygiene status was taken into consideration [34].

The degree of glycemic control, as indicated by the HbA1c level, is claimed to be an important variable in the relationship between DM and periodontal diseases. In another study, HbA1c values were shown to be significantly associated with severe periodontal destruction (CAL ≥ 5 mm), but not with other periodontal parameters [35]. In the present study, patients with T2DM and good glycemic control (HbA1c < 7) showed significantly better mean BOP indexes compared with patients who had poor glycemic control (HbA1c **≥** 7). In contrast, among patients with T2DM, comparison of periodontal destruction (CAL ≥ 5 mm) and number of teeth did not reach statistical significance between the metabolic groups (good and poor glycemic control). Accordingly, patients with well-controlled DM could have periodontal disease, just as those with poorly controlled DM could have a healthy periodontium [11].

Smoking is a known risk factor for periodontal disease [36]. In this study, the effect of current smoking did not reach statistical significance when compared to former and never smokers in the T2DM group. Moreover, statistical analysis failed to show significance between currently smoking controls and formerly and never smoking controls, except in the percentage of controls with severe periodontal destruction (CAL ≥ 5 mm). This might suggest that smoking was not a significant factor, which is consistent with recent literature [11]. Nevertheless, the findings in this study may be explained by the small number of smokers in both the T2DM and control groups (17.3 % and 11.5 %, respectively).

Finally, the estimated mean BOP index and severe periodontal destruction (CAL ≥ 5 mm) values were significantly related to poor oral hygiene level (PI > 1) in both the T2DM and control groups. This is consistent with the literature [34]. In contrast, number of teeth was not related to the PI in either the T2DM group or control group, which again may be attributable to the small numbers with a PI < 1 in each group.

### Genetic and microbiologic findings

As shown in Table 2, patients with T2DM hadworse periodontal conditions when compared with healthy controls. Therefore, intrinsic factors probably exerted an additional influence, and in support of this, there is evidence in the literature that periodontal health may be affected by polymorphisms in the interleukin 1 genotype [13, 37–39]. Indeed, it has been shown that specific interleukin 1 genotypes could increase the risk of tooth loss by 2.7 times [40]. In an attempt to explain the worse dental statuses of patients with T2DM, we therefore compared periodontal interleukin 1 genotypes between patients with and without T2DM to discover whether patients with T2DM were more likely to be positive for interleukin 1 genotypes. However, statistical analysis failed to detect any significant differences with respect to genetic groups A to D, suggesting that the worse dental status of patients with T2DM was not related to the interleukin 1 genotype. This is consistent with the work of Lopez et al., who showed that there were no statistically significant differences between patients with T2DM and metabolically healthy controls with respect to *IL1A*(−889), *IL1B*(+3954), and *IL1RN* genotypes [37]. In our study, 32 % of the 104 participants belonged to groups C and D, which corresponds to the rate of 30–35 % reported for Europeans [13, 37, 39, 41].

Analysis of the selected subgingival microbiota resulted in very similar results for both patients with T2DM and controls across profiles 1 to 8. This is consistent with existing research, which failed to show a significant difference in the subgingival plaque of participants with and without diabetes [21]. In contrast, significantly more patients with T2DM have been shown to harbor *P. gingivalis* when compared with nondiabetic controls [42]. However, in that study, the recruited patients with T2DM had more extensive periodontal disease than the control subjects, and it is likely that the increased levels of *P. gingivalis* were due to increased periodontitis rather than the presence of T2DM itself [12].

### Limitations

This study has some limitations. First, patients were recruited from an outpatient setting of a single university hospital. Therefore, the patients might not be representative for the whole population. Second, only short-term findings were recorded, and we cannot exclude completely that long-term evaluation may have produced different findings.

## Conclusions

Within the limitations of this study, it may be stated that patients with T2DM had worse oral hygiene and higher severity of periodontitis. Moreover, there were no differences with respect to the periodontal interleukin 1 genotype or selected subgingival microbiota between patients with T2DM and healthy controls. Therefore, we conclude that the dental plaque level remains the major contributory factor to progressive periodontitis in patients with T2DM. In contrast, polymorphisms of the interleukin 1 gene or differences in oral microbiota seem to play a subordinate role.

## Abbreviations

BMI: body mass index
BOP: bleeding on probing index
CAL: clinical attachment loss
DM: diabetes mellitus
HbA: hemoglobin A
HIV: human insufficiency virus
IL: interleukin
PCR: polymerase chain reaction
PPD: pocket probing depth
SPSS: Statistical Package for Social Sciences program
T1DM: type 1 diabetes mellitus
T2DM: type 2 diabetes mellitus
WHO: World Health Organisation
